# Long-term memory effects of an incremental blood pressure intervention in a mortal cohort

**DOI:** 10.1093/biomtc/ujaf176

**Published:** 2026-02-03

**Authors:** Maria Josefsson, Nina Karalija, Michael J Daniels

**Affiliations:** Department of Statistics, USBE, Umeå University, SE-90187, Umeå, Sweden; Center for Functional Brain Imaging, Umeå University, SE-90187, Umeå, Sweden; Department of Integrative Medical Biology, Umeå University, SE-90187, Umeå, Sweden; Department of Statistics, University of Florida, Gainesville, FL 32611, United States

**Keywords:** BART, dropout, G-computation, LDART, survival, truncation by death

## Abstract

In the present study, we examine long-term population-level effects on episodic memory of an intervention over 15 years that reduces systolic blood pressure in individuals with hypertension. A limitation with previous research on the potential risk reduction of such interventions is that they do not properly account for the reduction of mortality rates. Hence, one can only speculate whether the effect is due to changes in memory or changes in mortality. Therefore, we extend previous research by providing both an etiological and a prognostic effect estimate. To do this, we propose a Bayesian semi-parametric estimation approach for an incremental threshold intervention, using the extended G-formula. Additionally, we introduce a novel sparsity-inducing Dirichlet prior for longitudinal data, that exploits the longitudinal structure of the data. We demonstrate the usefulness of our approach in simulations, and compare its performance to other Bayesian decision tree ensemble approaches. In our analysis of the data from the Betula cohort, we found no significant prognostic or etiological effects across all ages. This suggests that systolic blood pressure interventions likely do not strongly affect memory, either at the overall population level or among individuals who would remain alive under both the natural course and the intervention (the always survivor stratum).

## 1 Introduction

Hypertension is a well-established risk factor for cognitive impairment and dementia (Livingston et al., 2020). However, the extent to which antihypertensive treatment can enhance cognitive abilities remains poorly understood, partly due to its long-term nature. Cognitive changes caused by hypertension likely develop over decades rather than years, making randomized clinical trials (RCTs) difficult to conduct (Iadecola et al., 2016). In this study, we extend previous research by examining the long-term population-level effects of an intervention that reduces systolic blood pressure (sBP) in individuals with hypertension. Our data come from the Betula Study, a representative prospective cohort study on aging, memory, and dementia (Nyberg et al., 2020). A key limitation of previous research is the insufficient consideration of altered mortality rates when estimating risk reduction. To address this, our study provides both etiological and prognostic intervention effect estimates. These estimates are critical for public health practice, offering a more comprehensive understanding of the intervention’s impact on cognitive health.

The G-formula (Robins, 1986) is commonly used to estimate causal effects in longitudinal settings where time-varying confounders influence both treatment and outcome. It simulates an intervention and, under a set of assumptions, yields causal effect estimates when these assumptions hold. This approach can provide valuable insights when RCTs are not feasible, and have been used previously for epidemiological research (eg, Zhou et al., 2020; Taubman et al., 2009). For a binary risk factor, this often involve setting the risk factor to a fixed value (Yes/No) (eg, Zhou et al., 2020), or by modifying a subject’s odds of receiving treatment through a hypothetical intervention based on incremental propensity scores (Kennedy, 2019). However, for a continuous risk factor, setting the treatment dose to a fixed value for all subjects is often considered to result in highly unrealistic scenarios with limited practical use. For example, a hypothetical intervention where all subjects in the population fix their sBP levels at 140 mm Hg would not take into account the natural variation that is also seen among non-hypertensive subjects. Another complicating factor for a continuous risk factor arises when only subjects meeting a diagnostic criteria are offered treatment. For instance, only subjects with hypertension would be offered antihypertensive agents to control their blood pressure levels.

Two approaches for hypothetical interventions on a continuous risk factor, which only intervene on subjects that do not reach a prespecified threshold, are the *threshold intervention* (Taubman et al., 2009) and the *representative intervention* (eg, Picciotto et al., 2012; Young et al., 2019). The former assigns the threshold value to all individuals who do not meet the prespecified target, while the latter assigns a value drawn from the distribution among those in the population meeting the target level. Hence, both approaches ensure treatment remains within a prespecified interval. Nevertheless, it is common for a significant proportion of the population to be unable to control their sBP at optimal levels, due to factors such as poor response to medications. One alternative approach in these settings is an *incremental intervention*, where a subject’s treatment is instead shifted downward (or upwards) by some prespecified function (Haneuse and Rotnitzky, 2013; Muñoz and Van Der Laan, 2012). However, these approaches are not valid for longitudinal data with dropout and deaths.

Attrition due to dropout and deaths is common in longitudinal aging studies, especially when subjects are followed for an extended duration. The G-formula can address both missing response and missing covariate data under an assumption of missing at random (MAR; Robins et al., 1995; Young et al., 2019; Kim et al., 2021). Further strategies for sensitivity analyses have been proposed in situations where the missingness is thought to be missing not at random (MNAR; Josefsson and Daniels, 2021; Josefsson et al., 2023. However, studying cognition after death is typically not defined and is not of interest, leading to consideration of a *mortal cohort* approach (Dufouil et al., 2004). Mortal cohort inferences truncate subjects’ outcomes after death. The first approach, *partly conditional inference* (PCI), conditions on the sub-population that is still alive at the end of follow-up (Kurland and Heagerty, 2005; Wen et al., 2018; Wen and Seaman, 2018). The second approach, *principal stratification*, conditions on the subpopulation that would survive through the end of follow-up regardless of treatment assignment, known as *the always survivor stratum* (Frangakis and Rubin, 2002; Frangakis et al., 2007). Although the two approaches have similarities, they differ with respect to the type of information they generate. PCI has been proposed for estimating non-causal associations and is particularly useful for prognostication, that is when the interest is in the health outcome among all individuals who are alive (eg, Taubman et al., 2009). In contrast, principal stratification is useful in situations where survival differs between treated and control groups and has been applied when the interest is in etiological effect estimates (eg, Hayden et al., 2005; Tchetgen Tchetgen, 2014; Wang et al., 2017).

A drawback of many approaches for estimating the Survivor Average Causal Effect is that they rely on the assumption of monotonicity for valid inference (see, eg, Josefsson and Daniels, 2021; Tan et al., 2021; Shardell and Ferrucci, 2018). This assumption implies that for a beneficial treatment, individuals who survive under treatment would also have survived in the absence of treatment. However, we consider this to be an implausible assumption in most, if not all, real-world settings. This is particularly true for populations with heterogeneous health profiles or competing risks of mortality. To address this limitation, Zaidi et al. (2019) proposed methods for sensitivity analyses that characterize causal effects at the population level while allowing for violations of monotonicity. In the context of clinical trials, Roy et al. (2008) and Lee et al. (2010) developed methods based on the less restrictive *stochastic monotonicity* assumption. This assumption relaxes standard monotonicity by allowing the probability that survival is higher under treatment than under control, conditional on covariates, to be less than one. However, these approaches are not valid for prospective data with time-varying treatments and time-varying confounding.

Our study extends previous research by providing both etiological and prognostic intervention effect estimates, which are both critical for public health practice, offering a more comprehensive understanding of the intervention’s impact. For estimation, we propose a Bayesian semi-parametric approach based on the extended G-formula (Robins et al., 2004). In addition, we introduce a novel sparsity-inducing Dirichlet prior designed for regression with longitudinal data, and demonstrate the usefulness of our approach in a simulation relative to other Bayesian tree ensemble approaches.

The paper is organized as follows. Section 2 introduces the intervention in a Bayesian setting; Section 3, the two types of mortal cohort inferences; Section 4, computational details and the sparsity-inducing Dirichlet prior; Section 5, a simulation study; Section 6, the Betula application; and Section 7, conclusions.

## 2 Methods

Consider a prospective cohort study where measurements on a set of variables are collected on each of $i = 1, 2, \ldots , n$ subjects over a specified follow-up period at $t=1, 2, \ldots ,T$ fixed time points. At time $t$, let $X_{it}$ denote the vector of time-varying confounders. We define $A_{it}$ as a continuous prognostic variable reflecting actionable treatment recommendations; for simplicity, we refer to $A_{it}$ as the “treatment” throughout. Denote the continuous outcome measure by $Y_{it}$, where interest in this study is the outcome measure at the last time point $T$. Furthermore, let $S_{it}=1$ indicate if a subject is alive at time point $t$; and $S_{it}=0$ otherwise. Let $R_{it}=1$ indicate if subject $i$ has completed the cognitive testing at time $t$; and $R_{it}=0$ otherwise. Let the observed history of a time-varying variable be denoted with an $\leftarrow$ and the future with an $\rightarrow$. At each time point $t$, we assume data are observed in the temporal ordering $S_{it}, R_{it}, X_{it}, A_{it}, Y_{it}$, and for each subject survival status is observed throughout follow-up, while the remaining variables are observed only during the period the subject actively participates in the study. Lower-case letters denote possible realizations of a random variable. We also write $H_{it}$ to denote the observed past history prior to the outcome variable at time point $t$. Specifically, $H_{it}=(\overleftarrow{Y}_{it-1}=\overleftarrow{y}_{it-1}, \overleftarrow{X}_{it}=\overleftarrow{x}_{it}, \overleftarrow{R}_{it}=\overleftarrow{r}_{it}, \overleftarrow{S}_{it}=1)$, with support on the set of possible histories $\mathcal {H}_{it}$. Note, $\overleftarrow{A}_{it}=\overleftarrow{a}_{it}$ is not included in $H_{it}$. In what follows, we suppress the subscript $i$ to simplify notation.

### 2.1 A hypothetical incremental threshold intervention

For the Betula data, we are interested in the long-term population-level effects of a 15-year intervention that reduces sBP in individuals with hypertension. As such, we consider two contrasting regimes: $g_*$, represents a setting where all individuals follow the intervention during the studied period. The natural course, $g_0 = \lbrace \overleftarrow{A}_{T} = \overleftarrow{a}_{T} \rbrace$, represents a setting in which all individuals follow their natural progression in the absence of an intervention. At each follow-up time point, we consider an intervention strategy in which treatment assignment is determined by a fixed rule based on the so-called the natural value of treatment (NVT), denoted by $A_{t}^{*}$. Following Young et al. (2014), $A_{t}^{*}$ is defined as the treatment measure that would have been observed at time $t$ if the intervention had been discontinued right before $t$. In particular, for the Betula data, we consider an intervention in which we intervene only on subjects whose $A_{t}^{*} > \tau$. The threshold $\tau$ is set by the researcher. We consider three cut-offs using values of 130 mmHg, 140 mmHg, and 150 mmHg for $\tau$.

We propose an intervention that assigns a new treatment value to individuals whose $A_{t}^{*} > \tau$. The new value is obtained by shifting $A_{t}^{*}$ downward by $\delta _t$. To quantify uncertainty in the intervention, we treat the shift $\delta _t$ as an unknown parameter and impose a prior distribution on it. The prior reflects the researcher’s beliefs about the plausible values that treatment can take under the intervention. Specifically, if $a_t^{*} \le \tau$, then the conditional intervention density is $p_*(a_{t} | \overleftarrow{y}_{t-1}, a_{t}^{*}, \overleftarrow{a}_{t-1}, \overleftarrow{x}_{t}, \overleftarrow{r}_{t}, \overleftarrow{s}_{t} = 1) = I\lbrace a_t = a_t^{*}\rbrace$. However, if $a_t^{*} > \tau$, then $p_*(a_{t} | \overleftarrow{y}_{t-1}, a_{t}^{*}, \overleftarrow{a}_{t-1}, \overleftarrow{x}_{t}, \overleftarrow{r}_{t}, \overleftarrow{s}_{t}=1) = p(\delta _t) I\lbrace a_t = a_t^{*} - \delta _t\rbrace$.

## 3 Intervention effects for mortal cohorts

In this section, we describe two types of mortal cohort inferences under the proposed intervention: PCI and *inference on the the always survivor stratum*. Let $\overleftarrow{Y}_T(g)$, $\overleftarrow{A}_T(g)$, $\overleftarrow{R}_T(g)$, $\overleftarrow{S}_T(g)$, and $\overleftarrow{X}_T(g)$ represent the counterfactual outcome, treatment, missingness, survival, and covariate histories associated with the regime $g$.

### 3.1 Partly conditional inference

Partly conditional models have been proposed to address truncation by death under hypothetical interventions. For these models, inference is conditional on the sub-population alive at a specific time-point, in our application being alive at end of follow-up (time $T$). Hence, inferences focus on what we call a *partly conditional intervention effect*: $\mathrm{PCIE} = E[Y_T(g_*) \mid S_T(g_*) = 1] - E[Y_T(g_0) \mid S_T(g_0) = 1]$.

Although the PCIE gives a crude comparison of differences in potential outcomes under the two regimes, which is useful for prognostication, it often implicitly compares potential outcomes for different underlying populations. For example, it is likely that an sBP intervention may affect survival rates; as such, the population surviving under the natural course will differ from the one surviving under the intervention.

The identification of the PCIE from longitudinal observational data relies on standard assumptions in causal inference: C1, consistency, C2, conditional exchangeability, and C3, sequential positivity. Additionally, we assume: C4, missing at random conditional on survival (MARS) for the outcome, treatment, and time-varying confounders. For a detailed discussion of these assumptions and their implications, see Supplementary Appendix A. Note that, for $g_{0}$, assumption C2 implies that, at each time point, treatment assignment is independent of potential outcomes given the observed past, which we denote as C2a. For $g_{*}$, this assumption states that the NVT at time $t$ has no direct effect on the outcome (or survival) except through future measurements of the treatment. An implication of this assumption is that the NVT is not itself a time-varying confounder (Richardson and Robins, 2013; Young et al., 2014). We denote this assumption as C2b. For the Betula data we perform sensitivity analyses to investigate robustness of results to violations of assumptions C2, C3, and C4, (see Section 6).

Under the assumptions C1, C2a, C3, and C4, the mean outcome at time $T$ under regime $g_{0}$, can be computed using the g-formula (Robins, 1986) conditioning on survival,


(1) \begin{eqnarray*}
& \int _{\overleftarrow{r}_{T}}\int _{\overleftarrow{x}_{T}}\int _{\overleftarrow{a}_{T}}\int _{\overleftarrow{y}_{T-1}} E[Y_T | \overleftarrow{y}_{T-1}, \overleftarrow{a}_{T}, \overleftarrow{x}_T, \overleftarrow{r}_{T}, \overleftarrow{s}_{T}=1] \\& \times \prod _{k=1}^T p(a_k | \overleftarrow{y}_{k-1}, \overleftarrow{a}_{k-1}, \overleftarrow{x}_{k}, \overleftarrow{r}_{k}, \overleftarrow{s}_{k}=1) \times \\& \qquad p(x_k | \overleftarrow{y}_{k-1}, \overleftarrow{a}_{k-1}, \overleftarrow{x}_{k-1}, \overleftarrow{r}_{k}, \overleftarrow{s}_{k}=1) \times \\& \qquad p(r_k | \overleftarrow{y}_{k-1}, \overleftarrow{a}_{k-1}, \overleftarrow{x}_{k-1}, \overleftarrow{r}_{k-1}, \overleftarrow{s}_{k}=1) \times \\& \qquad p(s_k | \overleftarrow{y}_{k-1}, \overleftarrow{a}_{k-1}, \overleftarrow{x}_{k-1}, \overleftarrow{r}_{k-1}, \overleftarrow{s}_{k-1}=1) \times \\& \qquad p(y_{k-1} | \overleftarrow{y}_{k-2}, \overleftarrow{a}_{k-1}, \overleftarrow{x}_{k-1}, \overleftarrow{r}_{k-1}, \overleftarrow{s}_{k-1}=1) \\& \times \, \displaystyle d\overleftarrow{y}_{T-1} d\overleftarrow{a}_T d\overleftarrow{x}_T d\overleftarrow{r}_T.
\end{eqnarray*}

In contrast, for the intervention, $g_{*}$, under assumptions C1, C2b, C3, and C4, the mean outcome at time $T$ can be computed using the *Extended g-formula* (Robins et al., 2004). The key difference is that $p(a_k | \overleftarrow{y}_{k-1}, \overleftarrow{a}_{k-1}, \overleftarrow{x}_{k}, \overleftarrow{r}_{k}, \overleftarrow{s}_{k}=1)$ in (1) is replaced by $\int _{a_{t}^{*}}p_{*}(a_k | \overleftarrow{y}_{k-1}, a^{*}_{k}, \overleftarrow{a}_{k-1}, \overleftarrow{x}_{k}, \overleftarrow{s}_{k}=1) \times p(a^{*}_k | \overleftarrow{y}_{k-1}, \overleftarrow{a}_{k-1}, \overleftarrow{x}_{k}, \overleftarrow{s}_{k}=1)$; details are given in Supplementary Appendix B.

The PCIE is then computed as the differences in expected outcomes under the two regimes $g_{0}$ and $g_{*}$. More details are given in Supplementary Appendix C.

### 3.2 Inference on the always survivor stratum

Conditioning on survival as described in Section 3.1 may introduce bias due to the fact that survival is a post-randomization event (Zhang and Rubin, 2003). Hence, the partly conditional approach in Section 3.1 cannot address etiological research questions in mortal cohorts. An alternative estimand is the causal effect on the subpopulation who would survive through the end of follow-up regardless of treatment regime (Frangakis and Rubin, 2002; Frangakis et al., 2007). Our goal here is to estimate the difference in expected potential outcomes between $g_{0}$ and $g_{*}$ in the always survivor stratum. That is, what we call a *survivors average intervention effect*: . Note, previous studies have shown a the link between reduced sBP and reduced all-cause mortality (Bundy et al., 2017). Hence, it is likely that an intervention targeting sBP not only may have an effect on cognition but also on mortality rates. In such situations, the PCIE would compare potential outcomes for different underlying populations. In particular, if subjects live longer under the risk-reducing intervention this population may include less healthy subjects who would die under the natural course, masking a possible intervention effect. The SAIE on the other hand, compare potential outcomes from the same population, namely subjects who would survive both when intervening on sBP and under the natural course. As such, it provides an estimate that adjusts for differences in mortality rates between the two regimes. In our application, we are interested in two types of effect estimates: a prognostic effect, the PCIE, and an etiological effect, the SAIE.

For identification of the SAIE, we need two additional assumptions.

**C5**  *Stochastic Monotonicity:*  $\Pr [S_{T}(g_*) = 1 \mid S_{T}(g_0) = 1] \ge \Pr [S_{T}(g_*) = 1 \mid S_{T}(g_0) = 0]$. In the current application, we impose an intervention that improves individuals’ health. As such, we assume the probability of survival under the intervention $g_{*}$ is higher among those subjects that would survive under the natural course $g_{0}$, compared to those who would not. We denote $\Pr [S_T(g) =1]$ by $\psi ^g$ for $g$ in $\lbrace g_0, g_*\rbrace$, and we assume $\psi ^{g_*} \ge \psi ^{g_0}$. We further assume $\Pr [S_T(g_*) = 1 \mid S_T(g_0) = 1] = \psi ^{g_*} + \lambda \left[\min \left\lbrace 1, \frac{\psi ^{g_*}}{\psi ^{g_0}}\right\rbrace - \psi ^{g_*}\right]$, where $\lambda$ is a sensitivity parameter (Roy et al., 2008; Lee et al., 2010). Note, $0\le \lambda \le 1$. If $\lambda =1$ this assumption corresponds to a *deterministic monotonicity* assumption where subjects who were to be alive under the observed regime $g_{0}$ would also be alive under the intervention $g_{*}$, i.e., $S_{T}(g_0) \le S_{T}(g_*)$. In contrast, when $\lambda = 0$, this corresponds to assuming independence between $S_{T}(g_0)$ and $S_{T}(g_*)$; we consider this assumption unlikely in the present application.

**C6**  *Difference in expectations of outcomes when comparing different strata*. $g \in \lbrace g_*, g_0\rbrace$, $g^{\prime } \in \lbrace g_*, g_0\rbrace$ and $g \ne g^{\prime }$, we assume, $E[Y_T(g) | S_T(g^{\prime }) = S_T(g)= 1] - E[Y_T(g) | S_T(g) = 1, S_T(g^{\prime }) = 0] = \Delta ^g$, where $\Delta ^g$ is a sensitivity parameter. We further assume that $\Delta ^g=\Delta ^{g^{\prime }}=\Delta$, where the difference in expectations of potential outcomes when comparing different strata is the same for the two contrasting regimes. Note, if $\Delta =0$ there is no difference in potential outcomes when comparing the always survivor strata to the strata where individuals were to live under regime $g$ but not under the contrasting regime $g^{\prime }$. In contrast, if $\Delta >0$ this implies a higher potential outcome is observed for the always survivor stratum. In the current application, we expect higher memory scores (better memory) for the always survivor stratum.

The identification details of the SAIE are given in Supplementary Appendix C. Briefly, under assumptions C1-C6, the SAIE is given by: $\mathrm{PCIE} + \Delta \left\lbrace \psi ^{g_*} + \lambda \left(U - \psi ^{g_*} \right) \right\rbrace \left(1 - \frac{1}{U} \right)$, where $U = \min \left\lbrace 1, \frac{\psi ^{g_*}}{\psi ^{g_0}} \right\rbrace$. The equation represents a location shift upwards from the PCIE for a beneficial intervention where we expect the participants to improve their health under the intervention compared to the natural course. Note, $\psi ^{g} = E_{(H_T,g)}\lbrace \Pr [S_T=1| H_T,\overleftarrow{A}_T=g]\rbrace$, for $g\in \lbrace g_*, g_0\rbrace$, are computed by marginalizing over the distributions of the set of temporally preceding variables using the g-formula and the extended g-formula.

## 4 A semi-parametric approach for estimation

In this section, we develop a Bayesian semi-parametric (BSP) modeling approach using *soft* Bayesian additive regression trees (BART) models to estimate each of the conditional distributions in (1) and (??) in Supplementary Appendix B. The *soft* BART model improves upon the traditional *hard* BART model by addressing the lack of smoothness inherent in ensemble approaches. To enhance valid inference, especially in scenarios with multiple time points and a large set of regressors, we extend the current *soft* BART algorithm by introducing a novel sparsity-inducing prior for longitudinal data. This longitudinal Dirichlet prior promotes parsimony by penalizing groups of predictors less likely to be important in longitudinal prediction settings. We refer to our model as a longitudinal Dirichlet additive regression trees (LDART) approach.

As in traditional (*hard*) BART models, the conditional mean function is given by: $\mu = \mu _{0} + \sum _{b=1}^B g(\mathbf {d};\mathcal {T}_b, \mathcal {M}_b)$, where $\mu _0$ is a global intercept, and the BART function is represented as a sum of regression trees. Here, $\mathbf {d}=\lbrace d_1, \ldots , d_P\rbrace$ denotes the set of predictors, $\mathcal {T}_b$ the structure of the $b$th tree, and $\mathcal {M}_b$ the corresponding terminal node parameters. Continuous outcomes are typically modeled with a Gaussian distribution, whereas binary outcomes are modeled using a probit link.

The regularization prior, controlling the complexity of the sum-of-trees, differ in two ways between the standard BART algorithm and *soft* BART. First, for *soft* BART, the path down the decision tree is probabilistic, where the sharpness of the decision is controlled by the bandwidth parameter. Thereby adapting to the smoothness level of the regression function even for low dimensional tree ensembles, a known shortcoming for (*hard*) BART models. Second, the $j$th predictor, from the set of $P$ predictors, is chosen according to a probability vector $\mathbf {q} = (q_1, \ldots , q_P)$, typically specified as uniform. In contrast, *soft* BART models incorporate a Dirichlet distribution for $\mathbf {q}$, such that $\mathbf {q} \sim \mathcal {D}\left( \alpha /P, \ldots , \alpha /P \right)$. The sparsity-inducing prior was first introduced for high dimensional settings (Linero, 2018), and can be extended to allow sparsity both within and between groups (Linero and Yang, 2018; Du and Linero, 2019). Determining the degree of sparsity in the model is done by placing a prior on $\alpha$, as such, $\frac{\alpha }{\alpha + \rho } \sim Beta\left(a, b \right)$. Default specifications for $a, b$, and $\rho$ is 0.5, 1, and $P$, respectively. This sparsity inducing prior is implemented for both *hard* and *soft* BART. We refer to BART models with this prior as Dirichlet additive regression trees (DART).

### 4.1 A grouping prior for longitudinal data

In a longitudinal setting, one may want to introduce a larger degree of sparsity for predictors collected at earlier time points, if they are thought to be less strongly associated with the response. Below we introduce a novel sparsity-inducing prior.

We consider a hierarchical structure where the predictors are divided into two groups: predictors measured at the *current* time point $t$ and predictors measured at *past* time points $k=1,\ldots ,t-1$. Moreover, baseline predictors, eg, age and sex, are grouped with the *current* predictors into one group, since we want to impose the same degree of sparsity on these predictors. Let $w$ refer to the probability of a predictor being from the set of *current* predictors, versus from the set of *past* predictors; we specify a beta prior, $w \sim Beta\left(a, b \right)$. For the *current* predictors, let $v^t_{j}$ refer to the probability of selecting the $j$th predictor from the set of $P_t$  *current* predictors ${\bf v}^t = (v^t_{1},\ldots ,v^t_{P_t}$); ${\bf v}^t$ is given a Dirichlet prior ${\bf v}^t \sim \mathcal {D}\left( \eta /P_t, \ldots , \eta /P_t \right)$. So a priori, the probability of selecting the $j$th *current* predictor to construct a given split is equal to $w \times v^t_{j}$. The *past* predictors are divided into $t-1$ groups, where $P_{k}$ is the size of group associated with time $k$. Let $u^k$ denote the probability of selecting the $k$th set of time-varying predictors, and let $v^k_{j}$ denote the probability of selecting the $j$th predictor in the $k$th set of time-varying predictors. To introduce sparsity related to proximity in time to the response we give ${\bf u}$ a grouping Dirichlet prior ${\bf u} \sim \mathcal {D}\left( \alpha _1/t-1, \ldots , \alpha _{t-1}/t-1 \right)$, where we determine the degree of sparsity in the model by placing a prior on $\alpha _k$ such that a larger degree of sparsity is obtained for smaller $k$’s (see details below). Similarly ${\bf v}^k$ is also given Dirichlet prior ${\bf v}^k \sim \mathcal {D}\left( \varphi ^k/P_k, \ldots , \varphi ^k/P_k \right)$. So a priori, the probability of selecting the $j$th predictor from the $k$th time point to construct a given split is equal to $(1-w) \times u^k \times v^k_{j}$.

For the hyperparameters $\eta$ and $\varphi ^k$ we use the default Beta hyperprior as implemented in DART. For $\alpha _j$ we specify a Beta hyperprior $\frac{\alpha _j}{\alpha _j + \rho _j} \sim Beta\left(c_j, d_j \right)$; we let $c_j$ vary as a linear function of time, set $d_j=1$, and $\rho _j=t$ for all $j$. To introduce sparsity related to proximity in time to the response we set $c_j = 1-\frac{(t - j)}{t-1} \times 0.5$. This results in ordered hyperpriors for the $\alpha _j$s, where $c_1=0.5$ for predictors measured at the first time point and $0.5{<}c_{j-1}{<}c_j{<} 1$ for $j{>}1$. That is, a higher degree of sparsity is introduced for predictors measured earlier in time and less sparsity for predictors measured closer in time to the response. The simulations in Section 5 shows the strong performance of these prior choices. Details about the posterior computations of the L-Dirichlet (LDART) prior are obtained in the Supplementary Appendix D.

The PCIE and SAIE use the algorithm described in Supplementary Appendix D, implemented in the R package GcompBART (Josefsson, 2025).

## 5 Simulation study

In this simulation study, we evaluate the performance of LDART and compare it to other BART specifications.

Data were generated according to a modified version of the Friedman’s five-dimensional test function (Friedman, 1991), mirroring a longitudinal study with five time-varying predictors and a continuous outcome. Specifically, $f(\overleftarrow{\mathbf {x}}_{i}) = \sum _{t=1}^T c_t \left[ 10 \sin (\pi x_{1,it} x_{2,it}) + 20 (x_{3,it} - 0.5)^2 + 10 x_{4,it} + 5 x_{5,it} \right],$, where

$Y_{iT} \sim \mathrm{Normal}\left\lbrace f(\overleftarrow{\mathbf {X}}_{i}), \sigma _T\right\rbrace$ , with $T=4$, $\sigma _T=10$, and $c_t$ determines the time-specific association between predictors and outcome.

The longitudinal predictors, $\overleftarrow{\mathbf {X}}_{i} = \lbrace \mathbf {X}_{i1}, \ldots , \mathbf {X}_{iT}\rbrace$, with $\mathbf {X}_{it} = (x_{1,it}, \ldots , x_{5,it})^{\prime }$, were generated as correlated uniform random variables. Specifically, for each individual $i$ and predictor $p = 1, \ldots , 5$, the sequence $(x_{p,i1}, \ldots , x_{p,iT})$ was generated to be correlated over time according to a multivariate normal distribution with mean zero and covariance matrix $\Sigma$. Here, the $(t,k)$ element of $\Sigma$ is given by $d_{tk}^2$. We specified $d_{t,t-1} = 0.4$, $d_{t,t-2} = 0.2$, and $d_{t,t-3} = 0.1$, reflecting stronger correlations for measurements closer in time. The resulting normal variables were then transformed to uniform margins using the standard normal cumulative distribution function, such that $x_{p,it} \sim \mathrm{Uniform}(0,1)$. Correlations were introduced only across time within each predictor, and all between-predictor correlations ($x_{p,it}$ and $x_{p^{\prime },it^{\prime }}$ for $p \ne p^{\prime }$) were set to zero.

We considered missingness in the form of dropout and assumed that approximately 15% of participants dropped out at each follow-up time point $t = 2, \ldots , T$. Dropout at time $j$ was generated as $\pi _j(\overleftarrow{\mathbf {x}}_{i}) = \mathrm{expit}\!\left[ h_j \sum _{t=1}^{j-1} \left(x_{1,it} + x_{2,it} + x_{3,it} + x_{4,it} + x_{5,it}\right) \right]$, where $\mathrm{expit}(x) = 1 / (1 + \exp (-x))$, $R_{ij} \sim \mathrm{Binomial}\lbrace \pi _j(\overleftarrow{\mathbf {x}}_{i})\rbrace$, and $\overleftarrow{h} = (0.75, 0.4, 0.25)$.

We simulated 2000 datasets of size $n = \left(250, 1000, 2000\right)$, and three different scenarios by varying $\overleftarrow{c}=\left(c_T,\ldots ,c_1\right)$. *Scenario A*: $\overleftarrow{c}=(1,0.75,0.5,0.25)$, reflecting a decreasing association over time between the predictors and the outcome; *Scenario B*: $\overleftarrow{c}=(1,0,0,0)$, where only predictors at the closest time point are associated with the outcome; *Scenario C*: $\overleftarrow{c}=(0.25,0.25,0.25,0.25)$, where a stable association with the outcome for all time points. For all scenarios we estimated $E_{(H_T, g_0)}\lbrace E[Y_T(g_0) \mid H_T, g_0]\rbrace$ using equation (1).

We compared the performance of our proposed approach (LDART) with two *hard* BART models: the standard BART algorithm (HBART) and BART with the sparsity-inducing Dirichlet prior (HDART) (as implemented in the R package BART; Sparapani et al., 2021), as well as two *soft* BART models: the standard SoftBart algorithm (SDART; as implemented in the R package SoftBart; Linero, 2022) and SoftBart incorporating only the predictors from the last time point in the model (SDART-l1). We specified 50 regression trees for each model. To evaluate their performance, we compared relative absolute bias, root-mean-squared error (RMSE), and 95% coverage probabilities (CPs). Unless stated otherwise, models used default priors, with 1200 warm-up and 2000 sampling iterations (thinning = 4) across 48 chains. Simulations were run in R 4.1 at the *High Performance Computing Center North*.

### 5.1 Simulation results

The simulation results are presented in detail in Table 1. For all scenarios and models, the absolute relative biases and RMSEs are smaller for the larger sample sizes, as expected. Moreover, the coverage probabilities are close to the nominal values for most scenarios, with CPs (%): 90.2–97.5. The *soft* BART models generally exhibited the smallest biases and RMSEs across the three scenarios. The best-performing model varied by scenario: in Scenario A, which reflects a decreasing association over time, LDART performed best; in Scenario B, where only predictors at the closest time point are associated with the outcome, SBART-l1 was the winner; and in Scenario C, characterized by a weak and stable association across all time points, SBART performed best. These results highlight the influence of the underlying data-generating mechanisms. Altough LDART was not the winner in Scenarios B and C, it was highly competitive, and that SBART-l1 achieved markedly smaller CPs in Scenarios A and C. The two *hard* BART models produces biases and RMSEs that are notably larger than the other models. In addition, they produce overly conservative credible intervals in many scenarios, with CPs close to 97.0%.

**Table 1:** Simulation results for estimating $E_{(H_T, g_0)}\lbrace E[Y_T(g_0) \mid (H_T, g_0)]\rbrace$ using (1) and the simulated data as described in Section 5 with 50 regression trees. For scenario A: ${c}=(1,0.75,0.5,0.25)$, B: ${c}=(1,0,0,0)$, and C: ${c}=(0.25,0.25,0.25,0.25)$. The results are presented in terms of absolute relative bias $\times 100$, root-mean-squared-error (RMSE) and 95% coverage probabilities as percentages [cp (%)]. The best-performing model for each scenario is highlighted.

	A			B			C		
	Bias	RMSE	CP (%)	Bias	RMSE	CP (%)	Bias	RMSE	CP (%)
*n* = 2000									
LBART	**0.86**	**0.39**	96.1	1.85	0.34	96.0	1.73	0.32	**95.4**
SBART	0.89	0.41	**95.4**	1.88	0.34	**95.0**	**1.70**	**0.31**	95.8
SBART-l1	0.98	0.44	92.1	**1.81**	**0.33**	94.7	1.85	0.34	92.8
HDART	0.99	0.46	95.8	1.99	0.37	96.1	2.02	0.37	96.4
HBART	1.00	0.45	96.4	2.12	0.39	97.0	1.94	0.35	97.5
*n* = 1000									
LBART	**1.20**	**0.55**	96.1	2.61	0.48	95.5	2.31	**0.42**	96.0
SBART	1.24	0.56	**95.2**	2.58	0.47	95.2	**2.28**	0.42	**95.6**
SBART-l1	1.22	0.56	94.0	**2.55**	**0.46**	**95.0**	2.47	0.45	92.6
HDART	1.32	0.60	96.6	2.79	0.51	96.4	2.66	0.49	96.2
HBART	1.33	0.61	96.0	2.87	0.53	96.5	2.71	0.49	97.3
*n* = 250									
LBART	**2.25**	**1.03**	**95.0**	4.97	0.91	**94.7**	4.67	0.85	92.6
SBART	2.33	1.06	95.0	4.84	0.88	94.5	**4.47**	**0.82**	**93.1**
SBART-l1	2.33	1.07	93.8	**4.70**	**0.86**	96.0	4.65	0.85	90.2
HDART	2.38	1.09	95.4	5.21	0.94	96.3	4.85	0.89	96.4
HBART	2.37	1.08	95.3	5.27	0.96	96.3	4.87	0.89	96.6

## 6 Analysis of the betula data

In the Betula study, we analyzed the long-term population-level effects of a 15-year intervention reducing sBP in individuals with hypertension, using the incremental threshold approach described in Sections 2–3. The analysis includes 800 participants from eight age cohorts (35–70 years at baseline) followed across four waves at five-year intervals.

Episodic memory was assessed using a composite score ranging from 0 to 76 (Josefsson et al., 2012). Baseline confounders were age, sex, and education, while time-varying covariates included body mass index, smoking, serum cholesterol, self-reported type 2 diabetes, and prior memory performance. Over the 15-year follow-up, 38% of participants dropped out, including 18% who either died or developed dementia. Dementia and death were treated jointly as a single terminating event, and results therefore reflect intervention effects among individuals who remained alive and cognitively healthy. See Table A1 for baseline summary statistics.

We consider an intervention where we assign sBP using the *incremental threshold intervention* described in Sections 2 and 3. The intervention loosely imply that, at each follow-up time point, sBP levels is shifted downwards by a prespecified value in individuals with hypertension, while no intervention is imposed on individuals without hypertension. See Fig. A1 for an illustration of the shift intervention using the Betula data. Specifically, we consider three cut-offs using values of 130 mmHg, 140 mmHg, and 150 mmHg for $\tau$. If a subject’s NVT at times $t=2,\ldots ,T$ is greater than $\tau$, then sBP is assigned by imposing a incremental shift on the NVT, and otherwise we do not intervene.

### 6.1 Priors for the shift and sensitivity parameters

Priors for the shift and sensitivity parameters are specified by the analyst and should reflect plausible values. Here, we specify a triangular prior for the shift parameter $\delta _t$, such that $\delta _t \sim \mathrm{Triangular}(L_{\tau }, 0, L_{\tau })$. The lower bound and mode are given by $L_{\tau }\approx -44$ mm Hg, corresponding to two standard deviations of baseline sBP, and the upper bound is 0. The intervention implies a negative shift from the NVT with between 0 and 44 mm Hg for individuals with a $NVT>140$ at each follow-up time point.

To estimate the SAIE, we also specify priors on the sensitivity parameters in Assumptions C5 and C6. We specify triangular priors for the sensitivity parameters $\Delta$ and $\lambda$. For $\Delta$, we assume $\Delta \sim \mathrm{Triangular}(0, U_{\Delta }, U_{\Delta }),$ with upper bound (and mode) set to $4.6\%$; $4.6\%$ corresponds to the estimated difference in baseline memory between those who are alive at time point $T$ and those who are dead, adjusting for age and sex. It is unlikely the difference between the strata would be bigger than this. We further assume $\lambda \sim \mathrm{Triangular}(0.5, 1, 1)$, where the upper bound corresponds to the deterministic monotonicity assumption while the lower bound reflects a moderate correlation between survival under the two regimes.

### 6.2 Results

We estimated the PCIE and the SAIE using LDART with priors on the sensitivity parameters as described above. For each of the 16 chains, we used a thinning of 100, discarded the first 300 iterations as burn-in, and kept 2400 posterior samples for inference. Note, with a thinning of 100, we ensured precision in terms of close to independent sampling for all age-cohorts. However, a much smaller number (10–20) would have been sufficient in most cases. We sampled pseudo data of size $N^{*}=10,000$ at each iteration for the MC integration. Convergence of the posterior samples was monitored using trace plots. Note, the memory outcome, a test score, is presented in %.

**Figure 1:**
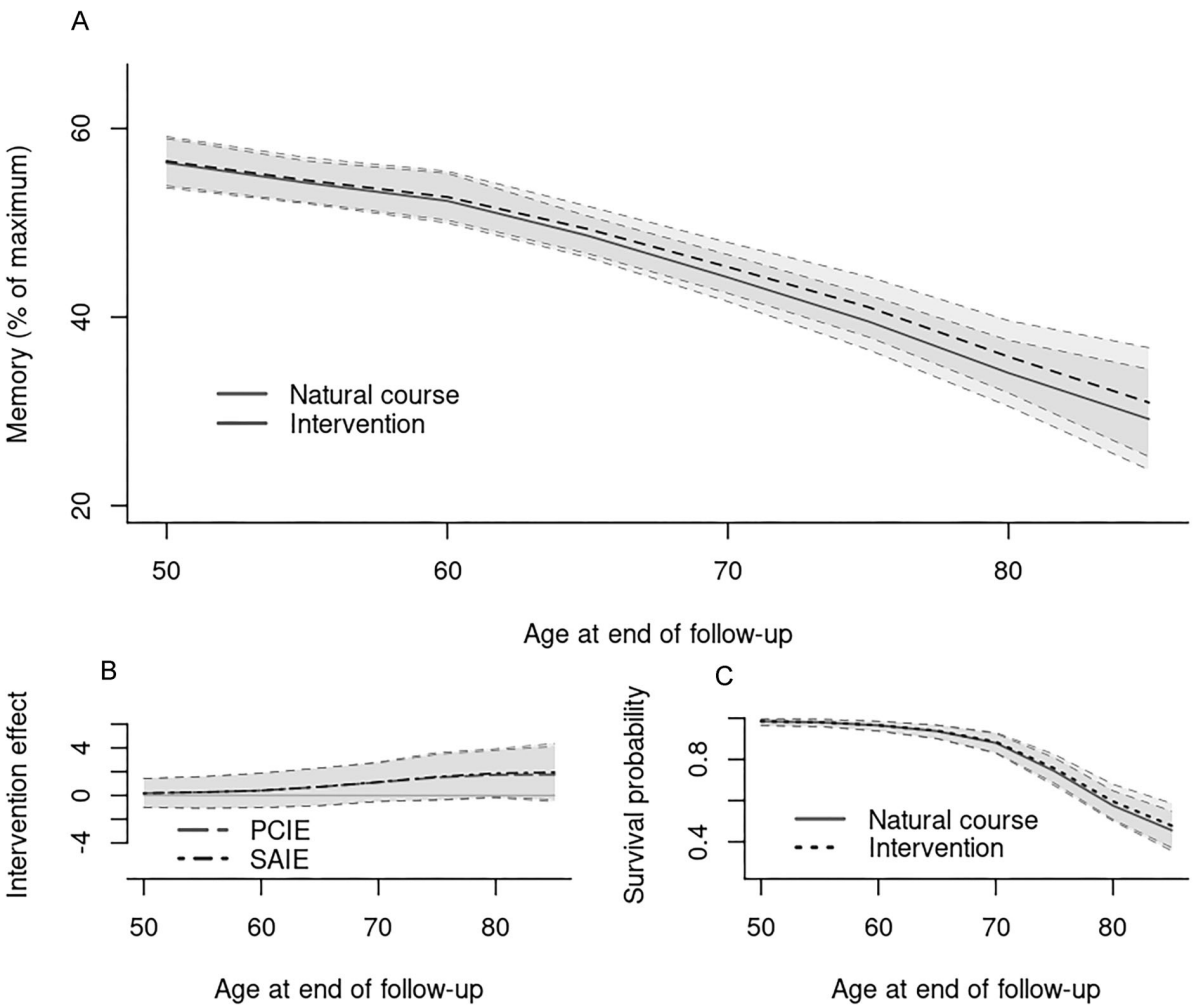
Results from the main analysis of Betula data using 130 mmHg as cut-off and default assumptions and sensitivity parameters as described in Section 6. Panel (A) Age-specific posterior mean memory at end of follow-up under the natural course (plain line) and under the hypothetical intervention (dotted line). Panel (B) Results for the PCIE and the SAIE. Panel (C) Posterior mean survival probabilities at end of follow-up under the natural course and the hypothetical intervention. All plots include 95% pointwise credible intervals.

Results for the different age-cohorts using 130 mmHg as cut-off are shown in Figure 1. We observe a consistent attenuation in effects as the threshold increases from 130 mmHg to 150 mmHg (see Supplementary Figure A2). In summary, posterior mean memory score at end of follow-up was attenuated with advancing age. This occurred both under the natural course and under the intervention, although to a lesser extent for the latter. Among subjects 70 and older, the shift in memory from the intervention corresponds to postponing memory decline approximately 2 years on average; however this effect is not significant. The results for the PCIE suggest that there is no long-term cognitive effects of monitoring sBP in this population (credible intervals cover 0 for all age-cohorts). We note however, that the effect is notably stronger for the older cohorts compared to middle-aged cohorts. The PCIE and SAIE estimates were similar. For the SAIE, this suggests no long-term effects within the always-survivor stratum, comprising individuals who would survive and remain free of dementia under both the natural course and the intervention. Hence, the non-significant results for the PCIE are not driven by differential mortality rates. We also note that only slightly higher survival probabilities were obtained under the intervention across age cohorts compared to the natural course, leading to rather small differences between the PCIE and the SAIE.

We performed sensitivity analyses to assess robustness to violations of assumptions C2–C6 (Supplementary Appendix E; Table A2). For clarity, analyses were stratified into middle-aged ($age_{iT}< 65$) and older ($age_{iT}\ge 65$) groups. Results were largely insensitive to alternative prior specifications for $\lambda$ and $\Delta$ (data not shown) and remained consistent when accounting for an unmeasured time-varying confounder. Excluding 61 subjects with limited propensity overlap slightly attenuated effects but did not alter conclusions. Assuming missing-not-at-random (MNARS) outcomes reduced posterior mean memory scores under both regimes, yet PCIE estimates remained stable. Results were similar when fitting alternative soft BART variants (S-DART, S-DART-l1) instead of LDART.

## 7 Conclusions

Using data from a prospective cohort study on aging, cognition, and dementia, we investigated population effects on episodic memory of an hypothetical incremental threshold intervention over 15 years that reduced sBP in individuals with hypertension. In our study, there were no significant prognostic or etiological effects, although effects were markedly stronger in older individuals. Nevertheless, these findings provide important insights into long-term blood pressure control and cognitive aging, suggesting that sBP interventions are unlikely to have a strong effect on memory, whether at the overall population level or among individuals who would remain alive and dementia free under both the natural course and the intervention. Importantly, the methodology applied in this study has broad applicability to interventional research in contexts where mortality is a major concern, including conditions as diverse as cancer, COVID-19, and congestive heart failure.

We propose a Bayesian semi-parametric approach for the proposed incremental threshold intervention, extending prior research in several ways. First, our approach introduces an incremental intervention for prospective cohort data with attrition, offering a viable alternative when clinical trials are not available or feasible to conduct. Second, we offer two types of mortal cohort inferences, providing both prognostic and etiological effect estimates, both of which are critical for public health practice, especially in older populations. Last, our modeling approach employs *soft* BART models with a novel sparsity-inducing prior for longitudinal data, promoting parsimony in longitudinal prediction settings and demonstrating good performance in simulations.

Concerns have been raised about principal stratification methods relying on assumptions that are often hard to justify in practice (eg, Feller et al., 2017). Monotonicity is one such assumption. Our approach relies on the less strong *stochastic monotonicity* assumption and uses sensitivity parameters that allow researcher to investigate sensitivity of results to this unverifiable assumption. However, a limitation with proposed principal stratification approach is that we only included age as a survival-predictive covariate for partial identification of the SAIE. In future work we will explore the possibility of incorporating more baseline covariates (see for example Roy et al., 2008; Tchetgen Tchetgen, 2014; Wang et al., 2017).

## Supplementary Material

ujaf176_Supplemental_Files Web Appendices, Supplementary results, and R code referenced in Sections 3, 4, and 6, are available with this paper at the Biometrics website on Oxford Academic. Web Appendices A–E provide details on assumptions, the extended G-formula, SAIE identification, computation (Dirichlet prior, G-computation), and supplementary Betula results. The R package GcompBART is available on https://github.com/m4ryjo/GcompBART (Josefsson, 2025).

## Data Availability

Betula data are available upon request and approval by the project’s Steering Group. See https://www.umu.se/en/research/projects/betula—aging-memory-and-dementia/.
