# Supplementary material for: Long-term memory effects of an incremental blood pressure intervention in a mortal cohort
Source: Biometrics. 2026 Feb 3;82(1):ujaf176. doi: 10.1093/biomtc/ujaf176 (PMC12865380; doi:10.1093/biomtc/ujaf176)
Supplement: ujaf176_Supplemental_Files — Web Appendices, Supplementary results, and R code referenced in Sections 3, 4, and 6, are available with this paper at the Biometrics website on Oxford Academic. Web Appendices A–E provide details on assumptions, the extended G-formula, SAIE identification, computation (Dirichlet prior, G-computation), and supplementary Betula results. The R package GcompBART is available on https://github.com/m4ryjo/GcompBART (Josefsson, 2025). [file ujaf176_supplemental_files.zip › Supplementary_material_Incremental_BP_Intervention.pdf]

Supplementary material for Long-term cognitive effects of an incremental blood pressure intervention in a mortal cohort

Josefsson, Maria, Karalija, Nina, & Daniels, Michael J.

A. Assumptions

A detailed description of the assumptions and their implications for identifying PCIE is provided below.

C1 *Consistency:* For $g \in (g_*, g_0)$ and $t = 1, \ldots, T$, if $\overleftarrow{A}_t = \overleftarrow{A}_t(g)$, then $\overleftarrow{Y}_t = \overleftarrow{Y}_t(g)$, $\overleftarrow{R}_t = \overleftarrow{R}_t(g)$, $\overleftarrow{S}_t = \overleftarrow{S}_t(g)$ and $\overleftarrow{X}_t = \overleftarrow{X}_t(g)$.

C2a *Conditional exchangeability under the natural course:* For g_0 and $t = 1, \ldots, T$, conditional on the history, the treatment and the potential outcome are independent, i.e.,

$$\overrightarrow{Y}_t(g_0) \perp\!\!\!\perp A_t | H_{t-1}, \overleftarrow{A}_{t-1} = \overleftarrow{a}_{t-1}$$

$$\overrightarrow{S}_{t+1}(g_0) \perp\!\!\!\perp A_t | H_t, \overleftarrow{A}_{t-1} = \overleftarrow{a}_{t-1}.$$

C2b *Conditional exchangeability for NVT interventions:* For g_*

$$(\overrightarrow{Y}_t(g_*), \overrightarrow{A}^*_t(g_*)) \perp\!\!\!\perp A_t | H_{t-1}, \overleftarrow{A}_{t-1} = \overleftarrow{a}_{t-1}$$

$$(\overrightarrow{X}_{t+1}(g_*), \overrightarrow{S}_{t+1}(g_*)) \perp\!\!\!\perp A_t | H_t, \overleftarrow{A}_{t-1} = \overleftarrow{a}_{t-1}.$$

This assumption states that under the intervention, the NVT at time t has no direct effect on the outcome, or survival, except through future measurements of the treatment.

An implication of this assumption is that the NVT is not itself a time-varying confounder (Richardson and Robins 2013; Young et al. 2014).

C3 *Sequential positivity:* Among subjects who have followed the regime g_* up to time t, there must be a positive probability of continuing to follow that regime at t, regardless of a participant's past information. That is, the conditional probability $p(a_t|\overleftarrow{y}_{t-1}, \overleftarrow{a}_{t-1}, \overleftarrow{x}_t, \overleftarrow{r}_t, \overleftarrow{s}_t = 1)$ must be > 0 and $p(\overleftarrow{y}_{t-1}, \overleftarrow{a}_{t-1}, \overleftarrow{x}_t, \overleftarrow{r}_t, \overleftarrow{s}_t = 1) \neq 0$, for all $\overleftarrow{a}_t$ consistent with regime g_*.

C4 *Missingness at follow-up:* For the outcome, treatment and time-varying confounders, we assume missing at random conditional on survival (MARS). In particular, for all $t > 1$,$p(y_t|\overleftarrow{a}_t, \overleftarrow{x}_t, r_t = 0, \overleftarrow{r}_{t-1}, \overleftarrow{s}_t = 1, \overleftarrow{y}_{t-1}) = p(y_t|\overleftarrow{a}_t, \overleftarrow{x}_t, \overleftarrow{r}_t = 1, \overleftarrow{s}_t = 1, \overleftarrow{y}_{t-1})$, and similarly for a_t and x_t.

B. The Extended G-formula

Below we provide details of the Extended G-formula as introduced in Section 4.2.

$$\int_{\overleftarrow{r}_t}\int_{\overleftarrow{x}_t}\int_{\overleftarrow{a}^*_t}\int_{\overleftarrow{a}_t}\int_{\overleftarrow{y}_{t-1}} E[Y_T|\overleftarrow{y}_{T-1},\overleftarrow{a}_T,\overleftarrow{x}_T,\overleftarrow{r}_T,\overleftarrow{s}_T=1]$$
$$\times\prod_{k=1}^{T} p_*(a_k|\overleftarrow{y}_{k-1},a^*_k,\overleftarrow{a}_{k-1},\overleftarrow{x}_k,\overleftarrow{s}_k=1)$$
$$\times\, p(a^*_k|\overleftarrow{y}_{k-1},d_{k-1},\overleftarrow{x}_k,\overleftarrow{s}_k=1)$$
$$\times\, p(l_k|\overleftarrow{y}_{k-1},\overleftarrow{a}_{k-1},\overleftarrow{x}_{k-1},\overleftarrow{r}_k,\overleftarrow{s}_k=1)$$
$$\times\, p(r_k|\overleftarrow{y}_{k-1},\overleftarrow{a}_{k-1},\overleftarrow{x}_{k-1},\overleftarrow{r}_{k-1},\overleftarrow{s}_k=1)$$
$$\times\, p(s_k|\overleftarrow{y}_{k-1},\overleftarrow{a}_{k-1},\overleftarrow{x}_{k-1},\overleftarrow{r}_{k-1},\overleftarrow{s}_{k-1}=1)$$
$$\times\, p(y_{k-1}|\overleftarrow{y}_{k-2},\overleftarrow{a}_{k-1},\overleftarrow{x}_{k-1},\overleftarrow{r}_{k-1},\overleftarrow{s}_{k-1}=1)$$
$$d\overleftarrow{y}_{t-1}d\overleftarrow{a}_t d\overleftarrow{x}_t d\overleftarrow{r}_t. \tag{A.1}$$

C. Identification of the SAIE under a hypothetical intervention based on the natural value of treatment

Identification of the SAIE under a hypothetical intervention based on the NVT under a stochastic Monotonicity assumption is presented below. First, for the two contrasting regimes g_0 and g_*, we have that the expected potential outcome for the principal strata becomes,

$$E[Y_T(g_*)-Y_T(g_0)\mid \overleftarrow{S}(g_0)=S_T(g_*)=1]=$$
$$PCIE+\Delta\left\{\psi^{g_*}+\lambda\left(U-\psi^{g_*}\right)\right\}\left(1-U^{-1}\right), \tag{A.2}$$

where $U = \min\left\{1, \frac{\psi^{g*}}{\psi^{g_0}}\right\}$. Identification of [A.2] is obtained by using the law of total probability (ltp) and some algebra. First, for the intervention regime g_*, and similarly for g_0, we have that

$$E[Y_T(g_*)) \mid S_T(g_*) = 1] =$$
$$E[Y_T(g_*) \mid S_T(g_0) = S_T(g_*) = 1] + \Pr[S_T(g_0) \neq 1 \mid S_T(g_*) = 1] \times$$
$$\left\{ E[Y_T(g_*) \mid S_T(g_0) = 1, S_T(g_*) \neq 1] - E[Y_T(g_*) \mid \overleftarrow{S}(g_0) = S_T(g_*) = 1] \right\}.$$

Using Assumption C6 and solving the above equation for $E[Y_T(g_*) \mid S_T(g_0) = S_T(g_*) = 1]$ we have that

$$E[Y_T(g_*) \mid \overleftarrow{S}(g_0) = S_T(g_*) = 1] =$$
$$E[Y_T(g_*) \mid S_T(g_*) = 1] + \Delta \times \{1 - \Pr[S_T(g_*) = 1 \mid S_T(g_0) = 1]\},$$

where Δ is a sensitivity parameter when comparing the difference in potential outcomes when comparing the principal strata to the strata where subjects were to live under the hypothetical intervention but not under the natural course. Hence,

$$E[Y_T(g_*) - Y_T(g_0) \mid \overleftarrow{S}(g_0) = S_T(g_*) = 1]$$
$$= E[Y_T(g_*) \mid S_T(g_*) = 1] + \Delta \times \{1 - \Pr[S_T(g_0) = 1 \mid S_T(g_*) = 1]\}$$
$$- E[Y_T(g_0) \mid S_T(g_0) = 1] - \Delta \times \{1 - \Pr[S_T(g_*) = 1 \mid S_T(g_0) = 1]\}$$
$$= E[Y_T(g_*) \mid S_T(g_*) = 1] - E[Y_T(g_0) \mid S_T(g_0) = 1] +$$
$$\Delta \times \{\Pr[S_T(g_*) = 1 \mid S_T(g_0) = 1] - \Pr[S_T(g_0) = 1 \mid S_T(g_*) = 1]\}$$
$$= PCIE + \Delta \times \{\Pr[S_T(g_*) = 1 \mid S_T(g_0) = 1] - \Pr[S_T(g_0) = 1 \mid S_T(g_*) = 1]\}$$

Moreover, by using C5 (Stochastic monotonicity) and Bayes theorem we have that $\Pr[S_T(g_*) = 1 \mid S_T(g_0) = 1] = \psi^{g_*} + \lambda(U - \psi^{g_*})$ and $\Pr[S_T(g_0) = 1 \mid S_T(g_*) = 1] = \frac{\psi^{g_0}}{\psi^{g_*}}[\psi^{g_*} + \lambda(U - \psi^{g_*})]$, where λ is a sensitivity parameter, and $0 \leq \lambda \leq 1$. Hence, the SAIE simplifies to (A.2).

For regimes $g \in (g_*, g_0)$, and H_T^g denote the history of the preceding variables up to time T under regime g, and using assumptions C1-C4, we have that

$$\begin{aligned} E[Y_T(g) \mid S_T(g) = 1] &= \frac{E[Y(g), S_T(g) = 1]}{\Pr[S_T(g) = 1]} \\ &= \frac{E_{H_T^g}[E(Y_T, S_T = 1 \mid H_T^g)]}{E_{H_T^g}[\Pr(S_T = 1 \mid H_T^g)]}. \end{aligned} \tag{A.3}$$

Under the natural course (g_0), $E_{H_T^{g_0}}[E(Y_T, S_T = 1 \mid H_T^{g_0})]$ is obtained by marginalizing over the distributions of the set of temporally preceding variables $H_T^{g_0}$ using Robins (1986)

g-formula. Thus, for g_0 we have that

$$E[Y_T(g_0), S_T(g_0) = 1] = E_{H_T^{g_0}}\{E[Y_T(g_0), S_T(g_0) = 1 \mid H_T^{g_0}]\}$$

$$= \int \dots \int E[Y_T(g_0) \mid \overleftarrow{S}_T = 1, \overleftarrow{Y}_{T-1} = \overleftarrow{y}_{T-1}, \overleftarrow{X}_T = \overleftarrow{x}_T, \overleftarrow{R}_T = \overleftarrow{r}_T]$$

$$\times \Pr[\overleftarrow{S}_T(g_0) = 1 \mid \overleftarrow{Y}_{T-1} = \overleftarrow{y}_{T-1}, \overleftarrow{X}_{T-1} = \overleftarrow{x}_{T-1}, \overleftarrow{R}_{T-1} = \overleftarrow{r}_{T-1}]$$

$$\times \prod_{k=1}^{T} p(Y_{k-1}(g_0) = y_{k-1} \mid \overleftarrow{Y}_{k-2} = \overleftarrow{y}_{k-2}, \overleftarrow{X}_{k-1} = \overleftarrow{x}_{k-1}, \overleftarrow{R}_{k-1} = \overleftarrow{r}_{k-1}, \overleftarrow{S}_{k-1} = 1)$$

$$\times p(A_k(g_0) = a_k \mid \overleftarrow{Y}_{k-1} = \overleftarrow{y}_{k-1}, \overleftarrow{X}_k = \overleftarrow{x}_k, \overleftarrow{R}_k = \overleftarrow{r}_k, \overleftarrow{S}_k = 1)$$

$$\times p(L_k(g_0) = l_k \mid \overleftarrow{Y}_{k-1} = \overleftarrow{y}_{k-1}, \overleftarrow{X}_{k-1} = \overleftarrow{x}_{k-1}, \overleftarrow{R}_{k-1} = \overleftarrow{r}_{k-1}, \overleftarrow{S}_k = 1)$$

$$\times p(R_k(g_0) = r_k \mid \overleftarrow{Y}_{k-1} = \overleftarrow{y}_{k-1}, \overleftarrow{X}_{k-1} = \overleftarrow{x}_{k-1}, \overleftarrow{R}_{k-1} = \overleftarrow{r}_{k-1}, \overleftarrow{S}_k = 1) d\overleftarrow{y}_{T-1} d\overleftarrow{a}_T d\overleftarrow{x}_T d\overleftarrow{r}_T$$

Exchangeability

$$= \int \dots \int E[Y_j(g_0) \mid \overleftarrow{S}_t = 1, \overleftarrow{Y}_{t-1} = \overleftarrow{y}_{t-1}, \overleftarrow{A}_t = a_t, \overleftarrow{X}_j = \overleftarrow{x}_j, \overleftarrow{R}_T = \overleftarrow{r}_T]$$

$$\times \Pr[\overleftarrow{S}_t(g_0) = 1 \mid \overleftarrow{Y}_{t-1} = \overleftarrow{y}_{t-1}, \overleftarrow{A}_{t-1} = a_{t-1}, \overleftarrow{X}_{t-1} = \overleftarrow{x}_{t-1}, \overleftarrow{R}_{T-1} = \overleftarrow{r}_{T-1}]$$

$$\times \prod_{k=1}^{T} p(y_{k-1} \mid \overleftarrow{Y}_{k-2} = \overleftarrow{y}_{k-2}, \overleftarrow{A}_{k-1} = a_{k-1}, \overleftarrow{X}_{k-1} = \overleftarrow{x}_{k-1}, \overleftarrow{R}_{k-1} = \overleftarrow{r}_{k-1}, \overleftarrow{S}_{k-1} = 1)$$

$$\times p(a_k \mid \overleftarrow{Y}_{k-1} = \overleftarrow{y}_{k-1}, \overleftarrow{A}_{k-1} = a_{k-1}, \overleftarrow{X}_k = \overleftarrow{x}_k, \overleftarrow{R}_k = \overleftarrow{r}_k, \overleftarrow{S}_k = 1)$$

$$\times p(l_k \mid \overleftarrow{Y}_{k-1} = \overleftarrow{y}_{k-1}, \overleftarrow{A}_{k-1} = a_{k-1}, \overleftarrow{X}_{k-1} = \overleftarrow{x}_{k-1}, \overleftarrow{R}_{k-1} = \overleftarrow{r}_{k-1}, \overleftarrow{S}_k = 1)$$

$$\times p(r_k \mid \overleftarrow{Y}_{k-1} = \overleftarrow{y}_{k-1}, \overleftarrow{A}_{k-1} = \overleftarrow{a}_{k-1}, \overleftarrow{X}_{k-1} = \overleftarrow{x}_{k-1}, \overleftarrow{R}_{k-1} = \overleftarrow{r}_{k-1}, \overleftarrow{S}_k = 1) d\overleftarrow{y}_{T-1} d\overleftarrow{a}_T d\overleftarrow{x}_T d\overleftarrow{r}_T$$

Consistency

$$= \int \dots \int E[Y_j \mid \overleftarrow{S}_t = 1, \overleftarrow{Y}_{t-1} = \overleftarrow{y}_{t-1}, \overleftarrow{A}_t = a_t, \overleftarrow{X}_j = \overleftarrow{x}_j, \overleftarrow{R}_T = \overleftarrow{r}_T]$$

$$\times \Pr[\overleftarrow{S}_t = 1 \mid \overleftarrow{Y}_{t-1} = \overleftarrow{y}_{t-1}, \overleftarrow{A}_{t-1} = a_{t-1}, \overleftarrow{X}_{t-1} = \overleftarrow{x}_{t-1}, \overleftarrow{R}_{T-1} = \overleftarrow{r}_{T-1}]$$

$$\times \prod_{k=1}^{T} p(y_{k-1} \mid \overleftarrow{Y}_{k-1} = \overleftarrow{y}_{k-1}, \overleftarrow{A}_{k-1} = a_{k-1}, \overleftarrow{X}_{k-1} = \overleftarrow{x}_{k-1}, \overleftarrow{R}_{k-1} = \overleftarrow{r}_{k-1}, \overleftarrow{S}_{k-1} = 1)$$

$$\times p(a_k \mid \overleftarrow{Y}_{k-1} = \overleftarrow{y}_{k-1}, \overleftarrow{A}_{k-1} = a_{k-1}, \overleftarrow{X}_k = \overleftarrow{x}_k, \overleftarrow{R}_k = \overleftarrow{r}_k, \overleftarrow{S}_k = 1)$$

$$\times p(l_k \mid \overleftarrow{Y}_{k-1} = \overleftarrow{y}_{k-1}, \overleftarrow{A}_{k-1} = a_{k-1}, \overleftarrow{X}_{k-1} = \overleftarrow{x}_{k-1}, \overleftarrow{R}_{k-1} = \overleftarrow{r}_{k-1}, \overleftarrow{S}_k = 1)$$

$$\times p(r_k \mid \overleftarrow{Y}_{k-1} = \overleftarrow{y}_{k-1}, \overleftarrow{A}_{k-1} = \overleftarrow{a}_{k-1}, \overleftarrow{X}_{k-1} = \overleftarrow{x}_{k-1}, \overleftarrow{R}_{k-1} = \overleftarrow{r}_{k-1}, \overleftarrow{S}_k = 1) d\overleftarrow{y}_{T-1} d\overleftarrow{a}_T d\overleftarrow{x}_T d\overleftarrow{r}_T$$

$$= E_{H_T^{g_0}}\{E[Y, S_T = 1 \mid H_T^{g_0}]\} = E[Y, S_T = 1].$$

A similar argument shows that $\Pr[S_T(g_0) = 1] = E_{H_T^{g_*}}\{\Pr[S_T(g_0) = 1 \mid H_T^{g_*}]\} = E_{H_T^{g_*}}\{\Pr[S_T = 1 \mid H_T^{g_*}]\} = \Pr[S_T = 1]$, and therefore,

$$\frac{E[Y(g_0), S_T(g_0) = 1]}{\Pr[S_T(g_0) = 1]} = E_{g_0}[Y \mid S_T = 1]. \tag{A.4}$$

Hence, it follows that (A.4) can be estimated using the g-formula conditioning on survival at a specific time point as described in (1) in the main text.

For regime g_*, $E_{H_T^{g_*}}[E(Y_T, S_T = 1 \mid H_T^{g_*})]$ is also obtained by marginalizing over the distributions of the set of temporally preceding variables $H_T^{g_*}$. However, the extended g-formula must be used for a intervention that depends on the NVT (Robins et al. 2004). As such, for regime g_* we have that

$$\begin{aligned}
&E(Y_T(g_*), S_T(g_*) = 1) = E_{H_T^{g_*}}[E(Y_T(g_*), S_T(g_*) = 1) \mid H_T^{g_*}] \\
&\quad = \int \dots \int E[Y_T(g_*) \mid \overleftarrow{S}_T = 1, \overleftarrow{Y}_{T-1} = \overleftarrow{y}_{T-1}, \overleftarrow{X}_T = \overleftarrow{x}_T, \overleftarrow{R}_T = \overleftarrow{r}_T] \\
&\qquad \times \Pr[\overleftarrow{S}_T(g_*) = 1 \mid \overleftarrow{Y}_{T-1} = \overleftarrow{y}_{T-1}, \overleftarrow{X}_{T-1} = \overleftarrow{x}_{T-1}, \overleftarrow{R}_{T-1} = \overleftarrow{r}_{T-1}] \\
&\qquad \times \prod_{k=1}^{T} p(Y_{k-1}(g_*) = y_{k-1} \mid \overleftarrow{Y}_{k-2} = \overleftarrow{y}_{k-2}, \overleftarrow{X}_{k-1} = \overleftarrow{x}_{k-1}, \overleftarrow{R}_{k-1} = \overleftarrow{r}_{k-1}, \overleftarrow{S}_{k-1} = 1) \\
&\qquad\quad \times p_*(a_k \mid \overleftarrow{Y}_{k-1} = \overleftarrow{y}_{k-1}, A_k^* = a_k^*, \overleftarrow{A}_{k-1} = \overleftarrow{a}_{k-1}, \overleftarrow{X}_k = \overleftarrow{x}_k, \overleftarrow{R}_k = \overleftarrow{r}_k, \overleftarrow{S}_k = 1) \\
&\qquad\quad \times p(A_k^*(g_*) = a_k^* \mid \overleftarrow{Y}_{k-1} = \overleftarrow{y}_{k-1}, \overleftarrow{X}_k = \overleftarrow{x}_k, \overleftarrow{R}_k = \overleftarrow{r}_k, \overleftarrow{S}_k = 1) \\
&\qquad\quad \times p(L_k(g_*) = l_k \mid \overleftarrow{Y}_{k-1} = \overleftarrow{y}_{k-1}, \overleftarrow{X}_{k-1} = \overleftarrow{x}_{k-1}, \overleftarrow{R}_{k-1} = \overleftarrow{r}_{k-1}, \overleftarrow{S}_k = 1) \\
&\qquad\quad \times p(R_k(g_*) = r_k \mid \overleftarrow{Y}_{k-1} = \overleftarrow{y}_{k-1}, \overleftarrow{X}_{k-1} = \overleftarrow{x}_{k-1}, \overleftarrow{R}_{k-1} = \overleftarrow{r}_{k-1}, \overleftarrow{S}_k = 1) d\overleftarrow{y}_{T-1} d\overleftarrow{a}_T^* d\overleftarrow{a}_T d\overleftarrow{x}_T d\overleftarrow{r}_T \\
&\quad = \dots
\end{aligned}$$

...

Exchangeability

$$= \int \dots \int E[Y_T(g_*) \mid \overleftarrow{S}_T = 1, \overleftarrow{Y}_{T-1} = \overleftarrow{y}_{T-1}, \overleftarrow{A}_T = a_T, \overleftarrow{X}_T = \overleftarrow{x}_T]$$

$$\times \Pr[\overleftarrow{S}_T(g_*) = 1 \mid \overleftarrow{Y}_{T-1} = \overleftarrow{y}_{T-1}, \overleftarrow{A}_{T-1} = a_{T-1}, \overleftarrow{X}_{T-1} = \overleftarrow{x}_{T-1}, \overleftarrow{R}_{T-1} = \overleftarrow{r}_{T-1}]$$

$$\times \prod_{k=1}^{T} p(y_{k-1} \mid \overleftarrow{Y}_{k-2} = \overleftarrow{y}_{k-2}, \overleftarrow{A}_{k-1} = a_{k-1}, \overleftarrow{X}_{k-1} = \overleftarrow{x}_{k-1}, \overleftarrow{R}_{k-1} = \overleftarrow{r}_{k-1}, \overleftarrow{S}_{k-1} = 1)$$

$$\times p_*(a_k \mid \overleftarrow{Y}_{k-1} = \overleftarrow{y}_{k-1}, A_k^* = a_k^*, \overleftarrow{A}_{k-1} = a_{k-1}, \overleftarrow{X}_k = \overleftarrow{x}_k, \overleftarrow{R}_k = \overleftarrow{r}_k, \overleftarrow{S}_k = 1)$$

$$\times p(a_k^* \mid \overleftarrow{Y}_{k-1} = \overleftarrow{y}_{k-1}, \overleftarrow{A}_{k-1} = a_{k-1}, \overleftarrow{X}_k = \overleftarrow{x}_k, \overleftarrow{R}_k = \overleftarrow{r}_k, \overleftarrow{S}_k = 1)$$

$$\times p(l_k \mid \overleftarrow{Y}_{k-1} = \overleftarrow{y}_{k-1}, \overleftarrow{A}_{k-1} = a_{k-1}, \overleftarrow{X}_{k-1} = \overleftarrow{x}_{k-1}, \overleftarrow{R}_{k-1} = \overleftarrow{r}_{k-1}, \overleftarrow{S}_k = 1)$$

$$\times p(r_k \mid \overleftarrow{Y}_{k-1} = \overleftarrow{y}_{k-1}, \overleftarrow{A}_{k-1} = \overleftarrow{a}_{k-1}, \overleftarrow{X}_{k-1} = \overleftarrow{x}_{k-1}, \overleftarrow{R}_{k-1} = \overleftarrow{r}_{k-1}, \overleftarrow{S}_k = 1) d\overleftarrow{y}_{T-1} d\overleftarrow{a}_T^* d\overleftarrow{a}_T d\overleftarrow{x}_T d\overleftarrow{r}_T$$

Consistency

$$= \int \dots \int E[Y_T \mid \overleftarrow{S}_T = 1, \overleftarrow{Y}_{T-1} = \overleftarrow{y}_{T-1}, \overleftarrow{A}_T = a_T, \overleftarrow{X}_T = \overleftarrow{x}_T, \overleftarrow{R}_T = \overleftarrow{r}_T]$$

$$\times \Pr[\overleftarrow{S}_T = 1 \mid \overleftarrow{Y}_{T-1} = \overleftarrow{y}_{T-1}, \overleftarrow{A}_{T-1} = a_{T-1}, \overleftarrow{X}_{T-1} = \overleftarrow{x}_{T-1}, \overleftarrow{R}_{T-1} = \overleftarrow{r}_{T-1}]$$

$$\times \prod_{k=1}^{T} p(y_{k-1} \mid \overleftarrow{Y}_{k-1} = \overleftarrow{y}_{k-1}, \overleftarrow{A}_{k-1} = a_{k-1}, \overleftarrow{X}_{k-1} = \overleftarrow{x}_{k-1}, \overleftarrow{R}_{k-1} = \overleftarrow{r}_{k-1}, \overleftarrow{S}_{k-1} = 1)$$

$$\times p_*(a_k \mid \overleftarrow{Y}_{k-1} = \overleftarrow{y}_{k-1}, A_k^* = a_k^*, \overleftarrow{A}_{k-1} = \overleftarrow{a}_{k-1}, \overleftarrow{X}_k = \overleftarrow{x}_k, \overleftarrow{R}_k = \overleftarrow{r}_k, \overleftarrow{S}_k = 1)$$

$$\times p(a_k^* \mid \overleftarrow{Y}_{k-1} = \overleftarrow{y}_{k-1}, \overleftarrow{A}_{k-1} = a_{k-1}, \overleftarrow{X}_k = \overleftarrow{x}_k, \overleftarrow{R}_k = \overleftarrow{r}_k, \overleftarrow{S}_k = 1)$$

$$\times p(l_k \mid \overleftarrow{Y}_{k-1} = \overleftarrow{y}_{k-1}, \overleftarrow{A}_{k-1} = a_{k-1}, \overleftarrow{X}_{k-1} = \overleftarrow{x}_{k-1}, \overleftarrow{R}_{k-1} = \overleftarrow{r}_{k-1}, \overleftarrow{S}_k = 1)$$

$$\times p(r_k \mid \overleftarrow{Y}_{k-1} = \overleftarrow{y}_{k-1}, \overleftarrow{A}_{k-1} = \overleftarrow{a}_{k-1}, \overleftarrow{X}_{k-1} = \overleftarrow{x}_{k-1}, \overleftarrow{R}_{k-1} = \overleftarrow{r}_{k-1}, \overleftarrow{S}_k = 1) d\overleftarrow{y}_{T-1} d\overleftarrow{a}_T^* d\overleftarrow{a}_T d\overleftarrow{x}_T d\overleftarrow{r}_T$$

$$= E_{H_T^{g_*}}[E(Y_T, S_T = 1 \mid H_T^{g_*})] = E(Y_T, S_T = 1).$$

Similarly as for g_0 we have that $\Pr[S_T(g_*) = 1] = E_{H_T^{g_*}}\{\Pr[S_T(g_*) = 1 \mid H_T^{g_*}]\} = E_{H_T^{g_*}}\{\Pr[S_T = 1 \mid H_T^{g_*}]\} = \Pr[S_T = 1]$, and therefore,

$$\frac{E[Y(g_*), S_T(g_*) = 1]}{\Pr[S_T(g_*) = 1]} = E_{g_*}[Y \mid S_T = 1]. \tag{A.5}$$

Hence, it follows that (A.5) can be estimated using the extended g-formula conditioning on survival at a specific time point as described in (A.1) in the main text. Moreover, by combining the results above we show that the SAIE become (A.2).

D. Computational details

Posterior computations

Similar to Linero and Yang (2018), we obtain simple conjugate posterior updates for (w, v^t, u, v^k) by implementing the Bayesian backfitting approach to sample from the posterior using the Markov chain Monte Carlo algorithm (Chipman et al. 2010). In particular, the full conditional for w is given by $w \sim Beta\,(a + m_{w_1}, b + m_{w_2})$, where m_{w_1} is the number of branch splits for the set of current predictors and m_{w_2} is the number of branch splits for all the sets of preceding time-varying covariates. Similarly, the full conditional of $\mathbf{v}^t$ and $\mathbf{v}^k$ are given by $\mathbf{v}^t \sim \mathcal{D}\left(\eta/P_t + m_{v_1^t}, \ldots, \eta/P_t + m_{v_{P_t}^t}\right)$ and $\mathbf{v}^k \sim \mathcal{D}\left(\eta/P_k + m_{v_1^k}, \ldots, \eta/P_k + m_{v_{P_k}^k}\right)$ respectively, where $m_{v_j^k}$ is the number of branch splits on the jth predictor in the set of predictors associated with time point $k = 1, \ldots, t-1$. Finally, the full conditional of $\mathbf{u}$ is given by $\mathbf{u} \sim D\left(\frac{\alpha_1}{t-1} + m_{u_1}, \cdots, \frac{\alpha_{t-1}}{t-1} + m_{u_{t-1}}\right)$, where m_{u_j} is the number of branch splits on the predictors in the jth set of time-varying covariates.

G-computation algorithm

The G-computation algorithm described below and is implemented in the R package, GcompBART (Josefsson 2025), which leverages Soft Bayesian Additive Regression Trees for flexible modeling of potential outcomes under truncation by death.

1. *Modeling the observed data*: For $t = 0, \ldots, T$, sample from the posterior distributions of the parameters of the conditional models for y_t, a_t, l_t, r_t, and s_t using LDART. The Bayesian bootstrap (Rubin 1981) is implemented to integrate over the distribution of baseline confounders without missingness.

2. *Pseudo data*: For each posterior sample of the LDART model parameters from Step 1, sequentially generate pseudo data, D_0 and D_*, of size N^p for the two contrasting regimes. Under the hypothetical intervention, pseudo NVT data for a_t are generated by drawing a_t^* from the observed posterior distribution, $a_t^* \sim N(\mu_{a_t}, \sigma_t^2)$, and computing $a_t = a_t^* + \delta_t I\{a_t^* \geq \tau\}$, where the shift parameter δ_t is drawn from its prior distribution.

3. *G-computation*: Implement G-computation, as described in Section 3, for the two contrasting regimes using the pseudo data, D_0 and D_*, and sensitivity parameters, $\overleftarrow{\delta}_t$ from Step 2. This yields posterior estimates, $\hat{\mu}^{g_*}$ and $\hat{\mu}^{g_0}$, of $E_{H_T^{g_*}}\{E[Y_T|S_T = 1, H_T^{g_*}]\}$ and $E_{H_T^{g_0}}\{E[Y_T|S_T = 1, H_T^{g_0}]\}$ respectively. For the SAIE, this step also involves computing the predicted survival at time T, $\hat{\psi}^g = \prod_{t=2}^{T} \hat{s}_t^g$, for the two contrasting regimes $g \in (g_*, g_0)$.

4. *Computation of the PCIE and the SAIE*: Using the pseudo data from the two

regimes, perform Monte Carlo integration to obtain one posterior sample of

$$PCIE = \frac{\sum_i \hat{\mu}_{iT}^{g*} s_{iT}^{g*}}{\sum_t^T \sum_i s_{iT}^{g*}} - \frac{\sum_i \hat{\mu}_{iT}^{g_0} s_{iT}^{g_0}}{\sum_t^T \sum_i s_{iT}^{g_0}}$$

and the

$$SAIE = PCIE + \Delta \left\{ \hat{\psi}^{g*} + \lambda \left(U - \hat{\psi}^{g*} \right) \right\} \left(1 - U^{-1} \right),$$

where $U = \min\left\{1, \frac{\hat{\psi}^{g*}}{\hat{\psi}^{g_0}}\right\}$, and one set of the sensitivity parameters Δ and λ is drawn for each posterior sample.

5. *Posterior repetition:* For each posterior draw of the model parameters obtained in Step 1, repeat Steps 2–4 to propagate parameter uncertainty through the computation of the posterior quantities of interest.

E. Supplementary Betula results

Table A1: Baseline demographics for the middle-aged and old group in the Betula study. Continuous variables are presented as mean ± SD and dichotomous variables as n (%).

	Middle aged	Older
Age	42.5 ± 5.6	62.5 ± 5.6
Male	0.46 ± 184	0.50 ± 198
Education	12.6 ± 3.8	8.5 ± 3.2
sBP	124.8 ± 15.6	147.7 ± 22.0
Smoker	230 (0.57%)	212 (0.53%)
BMI	24.3 ± 3.3	25.7 ± 3.5
Cholesterol	52.0 ± 19.2	60.8 ± 19.3
Diabetes type 2	6 (0.01%)	28 (0.07%)
Episodic memory	0.53 ± 0.12	0.42 ± 0.11

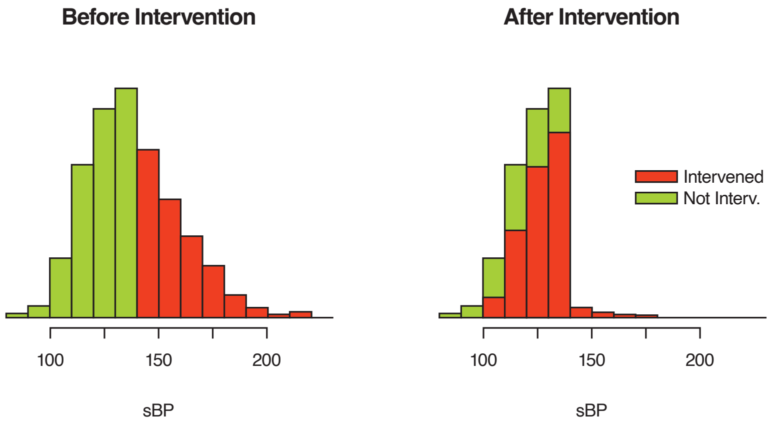

Figure A1: Illustration of the shift intervention using the Betula data. Only subjects with a sBP > 140 are intervened upon and δ is the shift parameter.

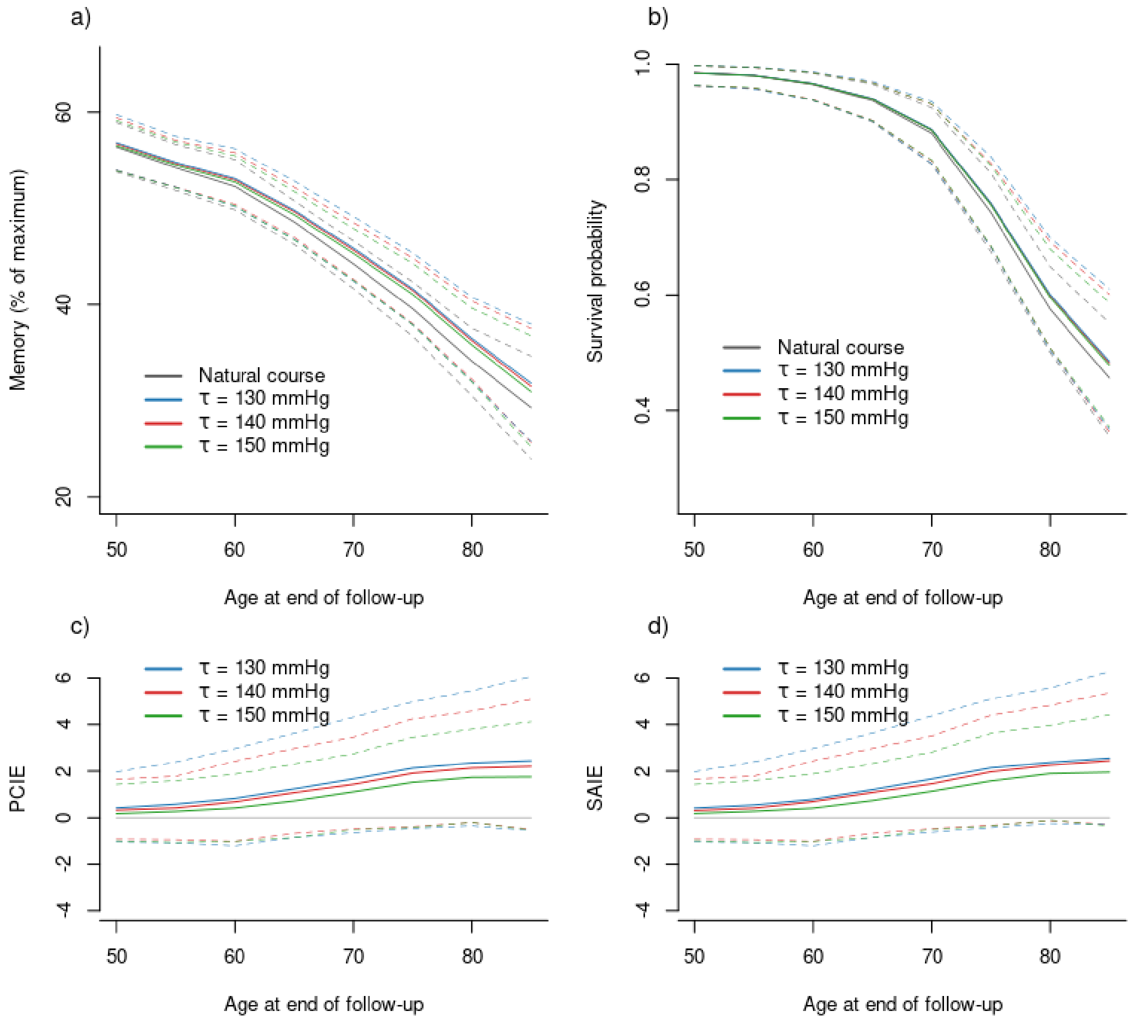

Figure A2: Results from the main analysis of Betula data using three cut-offs for τ and default assumptions and sensitivity parameters as described in Section 6. Panel a) Age-specific posterior mean memory at end of follow-up under the natural course and interventions. Panel b) Posterior mean survival probabilities at end of follow-up under the natural course and the hypothetical intervention. Panel c) Results for the PCIE. Panel d) Results for the SAIE. All plots include 95% pointwise credible intervals.

Sensitivity analyses

Below we provide details of the sensitivity analyses to investigate robustness of results to violations of the assumptions C2-C4 and other BART specifications.

Unmeasured confounding: We implement a simulation procedure to account for an unmeasured time-varying confounder missing for time-points $k > l$. In the Betula study, measures for cholesterol are obtained only for the first two time points; hence, it is a possible unmeasured confounder for $k > 2$. In the first step, U_k, for $k > 2$, is drawn from an estimate of the conditional distribution of $p(u_k \mid H^g_{u_k})$. Here, we assume $p(u_k \mid H^g_{u_k}) = p(u_2 \mid H^g_{u_2})$, where U_2 is the last observed time point for U_k and $H^g_{u_2} = (\overleftarrow{x}_2, \overleftarrow{r}_2, a_1, y_1)$ is the history for U_2. To obtain a plausible fit for the conditional distribution of $p(u_k \mid H^g_{u_k})$, this involves first obtaining a fit for $p(u_2 \mid H^g_{u_2})$ using LDART, then plugging $H^g_{u_k}$ into the LDART model (for u_2), and, finally, drawing realizations for U_k from the corresponding conditional distribution. Once we obtain realizations of U_k for $k > 2$, we then fit the (extended) g-formula to the full data, and estimate the intervention effect as described in Section 4. Since $H^g_{u_2}$ and $H^g_{u_k}$ corresponds to different number of predictors, we incorporate only a subset of the predictors for $H^g_{u_k}$. In particular, we include $(r_k, l_k, y_{k-1}, a_{k-1}, l_{k-1}, r_{k-1})$ as well as baseline covariates. Note, u_{k-1} is a subset of l_{k-1} and is observed for $k = 3$ and must first be estimated for $k > 3$. Since a single estimate corresponds to just one realization from the distribution of U_k, we simulate multiple draws (say 5) of U_k for $k > 2$, and an overall estimate of the intervention effect is obtained by averaging over the single estimates.

Positivity violations: To assess robustness of results to positivity violations (C3) we discard subjects with insufficient overlap. To identify these subjects we implemented a generalized propensity score approach, where the propensity score models at times $t = 2, \ldots, T$ were fitted using LDART. Subjects whose sBP was above the given threshold were excluded if their generalized propensity score were out of the range of the generalized propensity score among subjects with sBP within optimal levels.

Missingness for the outcome: Previous studies indicate that the dropout may be

MNAR for the outcome (Josefsson et al. 2012). As such, we introduce a sensitivity parameter γ_t to identify the distribution for dropouts among survivors, to allow for deviations from the MARS assumption (C4). We assume $p(y_t|\overleftarrow{a}_t, \overleftarrow{x}_t, r_t = 0, \overleftarrow{s}_t = 1, \overleftarrow{y}_{t-1}) = p(y_t - \gamma_t|\overleftarrow{a}_t, \overleftarrow{x}_t, \overleftarrow{r}_t = 1, \overleftarrow{s}_t = 1, \overleftarrow{y}_{t-1})$ for all $t > 0$. Our prior belief is that $\gamma_t < 0$, reflecting a negative shift in memory score after a subject drops out. Here, the prior is specified as $\gamma_t \sim Triangular(L_{\gamma_t}, 0, L_{\gamma_t})$, where we assume the lower bound not to be bigger than the estimated difference in memory change from baseline to follow-up between dropouts and completers, when adjusting for age and sex. That is, $\hat{E}[EMS_{i2} - EMS_{i1}|age_{i1}, sex_{i1}, r_{i3} = 1] - \hat{E}[EMS_{i2} - EMS_{i1}|age_{i1}, sex_{i1}, r_{i3} = 0]$, where EMS_{it} is the episodic memory score at time t.

Other soft BART specifications: We compared the performance of our proposed approach (LDART) to the two *soft* BART models; the standard SDART algorithm (as implemented in the R package SoftBart) and SoftBart incorporating only the predictors from the last time point in the model (SDART-lag1).

Table A2: Results for the $PCIE$ from the sensitivity analyses of the Betula data using the proposed approach. MARS refers to the default settings as described in Section 7. *Unmeasured confounding* refers to a sensitivity analysis for assumption A2, *Positivity* refers to a sensitivity analysis for A3, and MNARS refers to a sensitivity analysis A4b. SDART refers to the standard SoftBART and SDART-lag1 referes to SDART incorporating only the predictors from the last time point. See Section 7.2 for further details.

	Middle aged	Older
MARS	0.6 (-0.4; 1.8)	1.8 (-0.2; 4.0)
Sensitivity analyses		
Unmeasured confounding	0.6 (-0.5; 1.7)	1.7 (-0.4; 3.9)
Positivity	0.4 (-0.5; 1.5)	1.4 (-0.6; 3.4)
MNARS	0.6 (-0.5; 1.7)	1.7 (-0.4; 3.9)
SDART	0.2 (-0.7; 1.3)	0.7 (-1.4; 3.2)
SDART-lag1	0.0 (-0.8; 1.1)	0.1 (-1.2; 1.9)
